# Thyroid disease awareness is associated with high rates of identifying subjects with previously undiagnosed thyroid dysfunction

**DOI:** 10.1186/1471-2458-13-351

**Published:** 2013-04-16

**Authors:** Gay J Canaris, Thomas G Tape, Robert S Wigton

**Affiliations:** 1 University of Nebraska Medical Center, College of Medicine, Omaha, NE, USA

**Keywords:** Health fair, Screening, Bias, Thyroid disease

## Abstract

**Background:**

Conventional screening for hypothyroidism is controversial. Although hypothyroidism is underdiagnosed, many organizations do not recommend screening, citing low disease prevalence in unselected populations. We studied attendees at a thyroid health fair, hypothesizing that certain patient characteristics would enhance the yield of testing.

**Methods:**

We carried out an observational study of participants at a Michigan health fair that focused on thyroid disease. We collected patient-reported symptoms and demographics by questionnaire, and correlated these with the TSH values obtained through the health fair.

**Results:**

794 of 858 health fair attendees participated. Most were women, and over 40% reported a family history of thyroid disease. We identified 97 (12.2%) participants with previously unknown thyroid dysfunction. No symptom or combination of symptoms discriminated between hypothyroid and euthyroid individuals. Hypothyroid and euthyroid participants in the health fair reported each symptom with a similar prevalence (p > 0.01), a prevalence which was very high. In fact, when compared with a previously published case-control study that reported symptoms, the euthyroid health fair participants reported a higher symptom prevalence (range 3.9% to 66.3%, mean 31.5%), than the euthyroid individuals from the case-control study (range 2% to 54%, mean 17.4%).

**Conclusions:**

A high proportion of previously undiagnosed thyroid disease was identified at this health fair. We initially hypothesized symptoms would distinguish between thyroid function states. However, this was not the case in this health fair screening population. The prevalence of reported symptoms was similar and high in both euthyroid and hypothyroid participants. Because attendees were self-selected, it is possible that this health fair that focused on thyroid disease attracted participants specifically concerned about thyroid health. Despite the lack of symptom discrimination, the much higher prevalence of hypothyroidism in this study (12%) compared with the general population (<2%) suggests that screening may be appropriate and effective in certain circumstances such as thyroid health fairs.

## Background

Health promotion and disease prevention continue to gain emphasis in the evolving U.S. health care system. Even the Centers for Medicare and Medicaid Services (CMS) have embraced screening. The annual wellness exam is now a covered Medicare benefit, and screening tests such as bone densitometry are prescribed quality measures for the Physician Quality Reporting Initiative (PQRI) [1,2].

Health Fairs are a frequent fixture in health promotion programs. The intent is to detect treatable diseases early to improve the health of the community. Health fairs have historically been part of community service programs, efficiently using resources to reach large numbers of people. More recently, health fairs have included more specialized screenings of certain diseases in focused populations [3,4]. Health fairs do have the potential for data collection, but this is rarely done in a systematic fashion. In part, this may be because health fair populations are not always representative of the general population. Health fair attendees tend to be older when compared with the general population, likely because older retirees have more time to participate in such programs. Health fairs also tend to attract more women than men, as is the case with health care in general [5-9].

Health fairs may offer screening and information on multiple medical topics, or focus on a certain disease. One specialized screening offered by some health fairs is testing for thyroid disease. The prevalence of thyroid dysfunction in the general population is estimated between 1 and 2% [10]. For the individual person affected with thyroid disease, the disorder affects almost every organ system in the body. Functional thyroid disease is divided into hypothyroidism (underactive thyroid) and hyperthyroidism (overactive thyroid). With the development of assays that accurately measure thyroid hormone levels, these categories have been further subdivided into overt and subclinical disease. The classification is a misnomer in that clinical symptoms are not what distinguish subclinical disease. Rather, subclinical disease is defined by an abnormal TSH but normal free T4 level. In overt disease, both the TSH and free T4 levels are abnormal. The vast majority of thyroid dysfunction is hypothyroidism [10-12], so that hypothyroidism is often the focus of screening programs such as health fairs.

Many people with thyroid problems such as hypothyroidism go undiagnosed [12]. Symptoms develop gradually and are so nonspecific [13], that thyroid disease is not suspected and individuals may not seek medical attention. Screening for thyroid disease would therefore seem appropriate. Yet, many organizations recommend against general population screening for thyroid dysfunction. The U.S. Preventive Services Task Force, for example, states that “the yield of screening is greater in certain high-risk groups”, but does not recommend for or against routine screening for thyroid disease in asymptomatic adults [14]. However, individual symptoms associated with hypothyroidism are often unrecognized as such, making it difficult to identify the “symptomatic adult” [13]. Several groups do support screening among higher risk groups, such as women and older individuals, to increase the yield of testing [15,16].

One of the authors (GJC) obtained permission to collect data at a thyroid screening health fair in Michigan. The initial goal was to study the symptoms of those with and without thyroid disease in order to identify characteristics which increase the likelihood of an individual to have thyroid disease. A secondary goal was to determine the yield of thyroid screening in this population as compared with previous studies. We hypothesized that certain self-reported patient characteristics would increase the likelihood of thyroid disease, thus directing higher yield thyroid screening in the future.

## Methods

“Thyroid Awareness Week” was sponsored by St. Mary’s Health Services of Grand Rapids, Michigan, in conjunction with Boots Pharmaceutical Company. As part of Thyroid Awareness Week, information on thyroid disease was dispersed through radio, television and the press. A local endocrinologist lectured on thyroid disease, and thyroid function test screening was offered following the lecture. The screening activities were available at multiple locations and various times throughout the week.

The Health Fair offered thyroid function blood testing to anyone who presented to one of seven thyroid screening sites. The blood test used was the third generation supersensitive thyroid stimulating hormone (Ciba-Corning Diagnostics Corporation, Automated Chemiluminescence System assay) for a $5.00 charge through the Health Fair. This TSH assay has a normal range of 0.4 – 5.5 μIU/ml. Thyroxine (free T4) levels were not offered by the health fair.

Permission was obtained to carry out an observational study of participants in this health fair focused on testing for thyroid dysfunction. Data was collected at the Thyroid Awareness Week screening locations using a self-administered questionnaire. Each adult participant, age 18 and older, was asked to complete the Thyroid Symptoms Questionnaire to assess symptoms and obtain demographic information. This questionnaire was developed by one of the authors and has been used in other studies [12,13]. The questionnaire asked about the presence and severity of symptoms traditionally associated with thyroid disease, and if these symptoms had changed over the preceding year. Demographic information included age and gender, medications, and personal and family history of thyroid disease. The questionnaire took between five and ten minutes to complete.

Informed consent was obtained from each individual who wished to participate in the screening and complete the questionnaire. Informed consent included permission to obtain the individual’s TSH result from the health fair in order to link the TSH level to the questionnaire responses. Approval of the project was obtained from the sponsors of Thyroid Awareness Week, St. Mary’s Hospital Institutional Review Committee, and from the Butterworth Hospital Research and Human Rights Committee (the first author’s affiliation at the time). Results of the TSH were provided to each participant with instructions to share the information, regardless of result, with their health care provider.

### Data and statistical analysis

Data were analyzed using SAS statistical software, (SAS Institute, Inc. Cary, North Carolina). We applied the chi square test to compare proportions. Thyroid function was defined by the normal range of the TSH assay, 0.4 – 5.5 μIU/ml, inclusive. Hypothyroidism was defined as a TSH > 5.5 μIU/ml, and hyperthyroidism as a TSH < 0.4 μIU/ml.

We used logistic regression to examine the effect on TSH levels of age and family history in combination with each symptom. Regression was run with age and family history and the 33 symptom variables entered as independent variables. TSH as the dependent variable was analyzed at two thresholds. We analyzed the data using two different TSH thresholds, (greater than 5.5 μIU/ml the upper limit of the normal range for this assay, and greater than 10 μIU/ml), to test the hypothesis that symptoms become useful only in more severe disease. This is a threshold that has been applied in other studies to explore potential differences in what may be biochemically more advanced disease [17-20]. We also evaluated TSH as a continuous variable. The association between thyroid function (as measured by TSH) and symptoms was measured using the Spearman Rank-Order Correlation. Pearson Correlation Coefficient and linear regression was used to evaluate the association between TSH and the number of symptoms reported by any individual. Due to multiple comparisons, the more conservative threshold of p < 0.01 was used as the criterion for statistical significance throughout this study.

## Results

858 people presented to one of the seven thyroid screening sites. Sixty-four individuals were excluded from analysis for the following reasons: 41 (4.8%) did not return the questionnaire and/or demographic sheet, 7 (0.8%) were under age 18, and 16 (1.9%) completed fewer than 75% of the questionnaire responses. This left 794 participants for analysis (Tables 1 and 2). More women than men participated and the mean age of participants (51.9 years) was greater than that of the general population (30.0 years), features which are common to health fairs in general.

**Table 1:** Demographics of evaluable subjects

	**Men**	**Women**	**Total**
Number (%) of participants	147 (18.5)	647 (81.5)	794 (100)
Age range (mean) in years	20 – 86 (56.6)	20 – 87 (50.9)	20 – 87 (51.9)
Number (%) reporting a family history of thyroid disease	44 (30.3)	290 (45.2)	334 (42.4)
Range of TSH values in μIU/ml	< 0.03 – 582	< 0.03 – 128	< 0.03 – 582
50th percentile (TSH in μIU/ml)			2.2
75th percentile (TSH in μIU/ml)			3.7
90th percentile (TSH in μIU/ml)			6.7
Number (%) of TSH < 0.4 μIU/ml	9 (6.1)	37 (5.7)	46 (5.8)
Number (%) of TSH > 5.5 μIU/ml	16 (10.9)	86 (13.3)	102 (12.8)
Number (%) of TSH > 10 μIU/ml	6 (4.1)	39 (6.0)	45 (5.7)
Number (%) of participants on thyroid medication	12 (8.2)	94 (14.5)	106 (13.4)
Number (%) on thyroid medication who are euthyroid	6 (50.0)	49 (52.1)	55 (51.9)

**Table 2:** Demographics by thyroid function*

**Demographic**	**Hyperthyroid TSH < 0.4 μIU/ml**	**Euthyroid TSH = 0.4–5.5μIU/ml**	**Hypothyroid TSH > 5.5 μIU/ml**
Age			
Range in years	25 – 78	20 – 87	23 -87
(Mean)	(56.6)	(51.0)	(55.1)
Gender			
Male	9 (1.1%)	122 (15.4%)	16 (2.0%)
Female	37 (4.7%)	524 (66.0%)	86 (10.8%)
Family history**			
Positive	21 (2.6%)	268 (33.8%)	45 (5.7%)
Negative	25 (3.1%)	373 (47.0%)	55 (6.9%)

*Percentages reported are the proportion of the health fair population (n = 794).

**Responses for Family history were not provided by seven (0.9%) participants.

Participants were categorized as hypothyroid, euthyroid or hyperthyroid using the supersensitive TSH assay with normal range 0.4 – 5.5 μIU/ml. People with a TSH level greater than 5.5 μIU/ml were classified as hypothyroid, and those with a TSH level less than 0.4 μIU/ml were classified as hyperthyroid. Free T4 levels were not available through the Health Fair to further subclassify thyroid function regarding subclinical disease. TSH levels ranged from undetectable (<0.03 μIU/ml) to 582 μIU/ml with the following distribution: the 50th percentile corresponded to a TSH of 2.2 μIU/ml, the 75th percentile to a TSH of 3.7 μIU/ml and the 90th percentile to a TSH of 6.7 μIU/ml.

One-hundred forty-eight people had an abnormal TSH in this study (Table 3). Fifty-one (34.0%) of the 148 had known thyroid disease, so were not in the therapeutic range on their current medication. The remaining ninety-seven previously undiagnosed individuals, 12.2% of the total screened population, were identified through this screening program as having thyroid disease. Most of the thyroid disease detected (79.4%) was hypothyroidism. Thirty-three of the ninety-seven (34.0%) who were previously undiagnosed had a TSH greater than 10 μIU/ml.

**Table 3:** Participants taking/not taking thyroid hormone by thyroid function*

**Hyperthyroid**		**Euthyroid**		**Hypothyroid**	
**TSH < 0.4 μIU/ml**		**TSH = 0.4 – 5.5 μIU/ml**		**TSH > 5.5 μIU/ml**	
**On Thyroid**	**Not on Thyroid**	**On Thyroid**	**Not on Thyroid**	**On Thyroid**	**Not on Thyroid**
26 (3.3%)	20 (2.5%)	57 (7.2%)	589 (74.2%)	25 (3.1%)	77 (9.7%)
Total 46 (5.8%)		Total 646 (81.4%)		Total 102 (12.8%)	

*Percentages reported are the proportion of the health fair population (n = 794).

Of the 794 evaluable participants, 108 (13.6%) reported being on a thyroid medication. (Two of these listed their thyroid medication as an over-the-counter herbal supplement rather than a prescription thyroid hormone replacement; consequently 106 was the denominator used in analysis.) Of those taking thyroid medication, 94 (88.7%) were women and 12 (11.3%) were men. Three of the 12 men had a TSH less than 0.4 μIU/ml so were over-replaced, that is, on too high of a dose of thyroid hormone replacement putting the person in the hyperthyroid range. Three were under-replaced, or on too low of a dose of thyroid hormone with TSH greater than 5.5 μIU/ml. Six were in the euthyroid range. Ninety-four (14.5%) women reported taking thyroid medication; 23 were over-replaced on their medication so in the hyperthyroid range, 22 were under-replaced so in the hypothyroid range, so that only 49 (52.1%) were biochemically euthyroid.

Among the 97 participants without a previously known diagnosis of thyroid disease, 77 (9.7%) were hypothyroid and 20 (2.5%) were hyperthyroid. Seventy-eight were women and 19 were men. Of the nineteen men, thirteen had hypothyroidism and six were hyperthyroid. Of the women, 64 were diagnosed with hypothyroidism and fourteen with hyperthyroidism. Regarding family history, 290 women and 44 men reported a family history of thyroid disease (Tables 1 and 2). The breakdown of family history among disease states showed that 41.8% of euthyroid individuals reported a positive family history as compared with 45.0% of hypothyroid individuals reporting family history and 45.7% of hyperthyroid individuals reporting family history (p value = 0.75). Having family history of thyroid disease did not distinguish between euthyroidism and thyroid dysfunction.

The reported prevalence of individual symptoms in this health fair population was high (Additional file 1). The prevalence with which an individual symptom was reported by participants with hypothyroidism was similar to the prevalence reported by those without hypothyroidism, (not statistically significantly different, p > 0.01). The symptom prevalence reported by health fair participants whether hypothyroid or euthyroid, exceeded the prevalence reported by euthyroid controls in a previously published case-control study [13]. Euthyroid health fair participants’ symptom prevalence ranged from 3.9% to 66.3% (mean 31.5%), in fact higher than in the previous case-control study of the general population where euthyroid individuals reported symptom prevalence between 2% and 54% (mean 17.4%) [13]. When broken down by whether or not the health fair participant was taking thyroid medication, hypothyroid individuals did not show significant differences. However, euthyroid individuals who were taking medication reported four symptoms at a significantly higher prevalence than individuals who were euthyroid without medication (Additional file 2.) These symptoms included dry skin, cold sensitivity and the cognitive symptoms (slow thinking and poor memory) at the p < 0.01 level. Hoarse voice, being more sensitive to cold than compared to the previous year, and constipation approached statistical significance.

**Figure 1:**
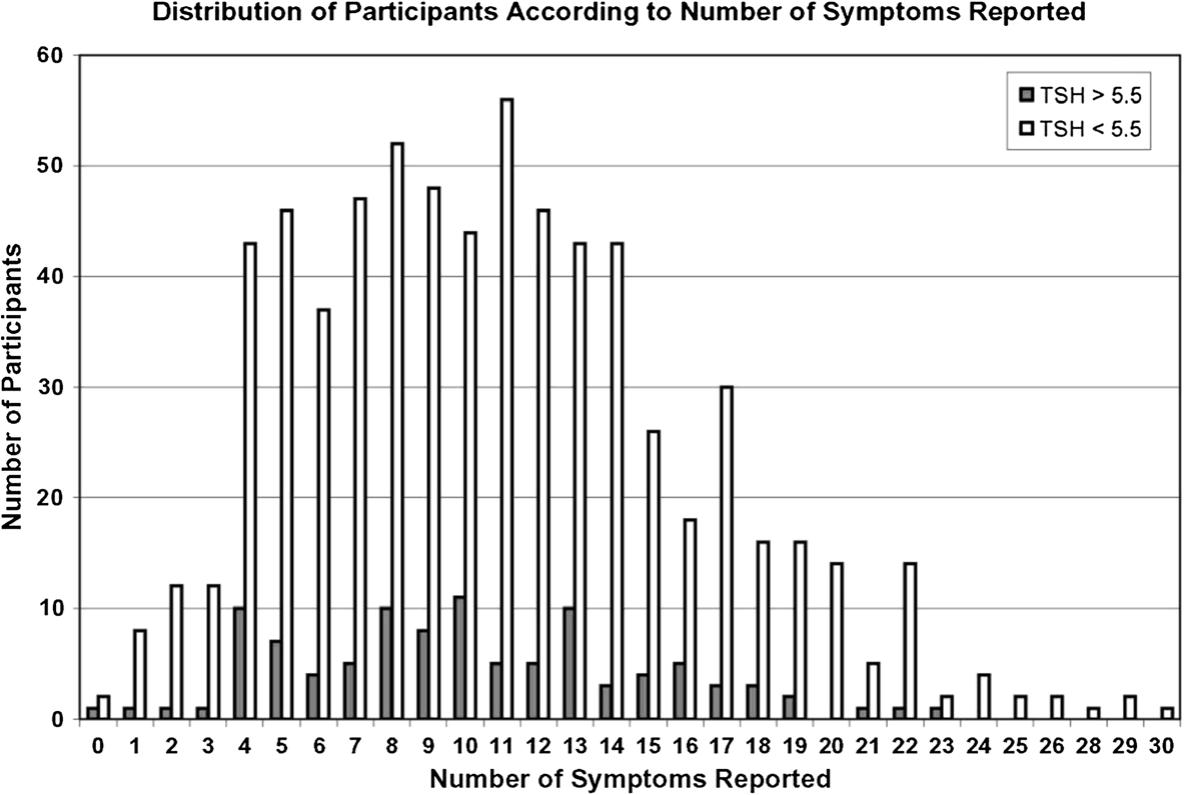
Distribution of participants according to number of symptoms reported.

Health fair participants reported a wide range of symptoms, from absolutely no symptoms to 30 symptoms listed on the questionnaire (Figure 1). The median number of symptoms reported by all health fair participants was ten, and mean number of symptoms was eleven.

Overall we found no clinically useful predictors of hypothyroidism among the symptoms. No individual symptom, number of symptoms, or specific combination of symptoms predicted TSH level. Logistic regression analysis showed no statistically significant predictors for hypothyroidism as defined by the upper limit of normal for the assay (TSH > 5.5 μIU/ml). Nor was there a cut point where symptoms became significant. That is, logistic regression showed no statistically significant predictors with TSH >10 μIU/ml. Additionally, we repeated the analysis with TSH level as a continuous variable, and it did not change this finding. Neither age nor family history correlated with TSH.

## Discussion and conclusions

The original goal of this project was to see if symptoms could distinguish between people with thyroid dysfunction and those who are euthyroid. We did not find this to be the case in this population. No symptom, nor group of symptoms, predicted hypothyroidism. Symptoms did not discriminate between disease states because both euthyroid and hypothyroid individuals reported similar symptom prevalence. Interestingly, the euthyroid people who presented to this thyroid screening health fair reported a high proportion of classic thyroid symptoms, higher than a group of euthyroid individuals in a previously published case-control study.

The secondary goal of the project (to determine the yield of thyroid screening in this population as compared with previous studies) found the yield of thyroid disease on testing this group of health fair attendees higher than previously reported in the literature [10]. This screening program identified 97 people (12.2%) with newly diagnosed thyroid dysfunction, 34% of which had a TSH greater than 10 μIU/ml. The 9.7% newly identified with hypothyroidism greatly exceeds the prevalence rates of hypothyroidism quoted in the literature (one to two percent of the general population) [10]. Our rates are higher even when the prevalence of subclinical hypothyroid is included in the comparison studies [11]. Interestingly, the rates of an elevated TSH found in our study are quite comparable to those reported in another health fair population [12].

There has been much discussion regarding traditional symptoms of thyroid disease and the utility of symptoms in detecting hypothyroidism. Such symptoms are not highly sensitive, though multiple investigators have tried to quantify symptoms to aid in diagnosis [13,21-23]. As well, symptoms may be too nonspecific to be helpful clinically, particularly in individuals with multiple comorbid conditions. Certainly individuals with co-morbid conditions manifest symptoms that could be attributed to thyroid disease. Information regarding comorbidities was not collected through the health fair, a limitation of our study.

The prevalence that symptoms were reported by all participants in this health fair study was considerably higher than the prevalence of symptoms previously reported in a case control study using the same symptoms questionnaire [13]. Every questionnaire symptom was reported more often by both hypothyroid and euthyroid participants in this screening study, than by the euthyroid controls in the previously published study. Several explanations are possible. Foremost, the thyroid health fair was advertised with promotional materials that highlighted the symptoms associated with thyroid disease. This certainly could have attracted people with more typical thyroid symptoms. Health fairs vary in focus [3,4,9,24,25], and as Lefebvre and colleagues showed, different screening offerings do attract different populations [9]. It is very likely that a health fair that advertises thyroid screening would attract people who are concerned about thyroid disease. Their concern may be related to symptoms attributable to thyroid disease, or to other factors such as family history of thyroid illness. People with a family history may be more aware of symptoms classically associated with thyroid dysfunction, and may want to participate in a thyroid screening. So, the higher prevalence of thyroid symptoms and of thyroid dysfunction found in our study may be explained by an increased awareness of personal symptoms and the high proportion of affected family members (42.4%). Symptom awareness was also likely enhanced by the educational activities during the thyroid health fair week. The increased prevalence of thyroid dysfunction found in our study may also be explained in part by the fact that health fairs typically attract individuals who are older and who are women [5-9]. This may have contributed to our findings since thyroid dysfunction, and hypothyroidism in particular, is more common in older people and is nearly ten times as common in women as in men [10]. The populations which are at higher risk for thyroid disease are over-represented in our health fair study (and at health fairs in general) as compared with the general population.

It is interesting that among euthyroid individuals, some symptoms were still reported significantly more often by euthyroid individuals taking thyroid medication than individuals that were naturally euthyroid. Despite correcting biochemical hypothyroidism with medication, these individuals still reported a higher prevalence of some classic hypothyroid symptoms than biochemically euthyroid individuals who had not had thyroid disease. Whether this reflects heightened awareness of classic symptoms of hypothyroidism, or if it suggests that medication does not completely reverse hypothyroid symptoms is unknown.

We also observed that almost half of the people identified as having abnormal thyroid function through this screening program were people taking thyroid medication but who were not euthyroid despite being on treatment. This finding is consistent with the literature [12], reinforcing the need for closer monitoring of patients on thyroid hormone replacement.

Our study was limited by free T4 levels not being available through this screening. We are therefore unable to comment on the amount of subclinical hypothyroidism as defined by an elevated ultrasensitive TSH but normal free T4 level. As well, there has been discussion that the target euthyroid TSH may actually be less than the upper limit of the TSH assay. While multiple studies have looked at clinical manifestations associated with these milder forms of thyroid dysfunction, it is certainly beyond the scope of this study. When viewed conservatively, that is at a cutoff commonly used to represent overt disease, the nearly six percent of the individuals we screened who had a TSH level elevated greater than 10 μIU/ml warrants consideration. Using a lower cutoff of TSH to define hypothyroidism would only enhance the yield of testing in our health fair population. Regarding symptoms, it is unknown but unlikely to affect results since there were no statistically significant differences in symptom reporting between euthyroid and hypothyroid individuals. We are also limited by having data from one point in time, as is the nature of health fairs. This does not allow us to know if any TSH results reflected transient abnormalities. However, it is unlikely that such deviations would favor abnormal or normal thyroid function in particular.

Thus, testing people who wish to be evaluated for thyroid disease can increase diagnostic yield. In our study, testing a self-selected population increased identification of previously unknown thyroid dysfunction from the less than 2% quoted for the general population to 12% in this health fair population. The observation that more disease is identified through a disease-specific health fair than is reported in the general population, we call the “health fair effect”. This may reflect education about traditional thyroid symptoms, impact of affected family members, or the characteristics of the people themselves having more risk factors such as age and female gender. While testing symptomatic people may increase yield, it did not allow discrimination between disease states in this particular population. Traditional thyroid symptoms were highly prevalent in all people who attended this health fair, so did not aid in identification of disease. It is possible that symptoms would be more discriminatory in a population that had not been educated on thyroid dysfunction, or in a setting outside of a health fair where people are motivated often by risk factors for the disease being screened. The application of symptoms to direct thyroid testing may be better suited to a clinical setting rather than a health fair, a possible direction for future research.

Thyroid screening does identify people who may otherwise go undiagnosed and thus untreated, and who may benefit from treatment because of the adverse effects of thyroid disease on multiple organ systems. But thyroid testing needs to be done in a setting where the chance of identifying disease is high enough to be beneficial. Testing a population with a likelihood of thyroid disease that is greater than the general population, is more desirable. The effect of this health fair was to draw a population more likely to have thyroid disease, for several possible reasons, and thus increase the yield of testing for thyroid disease. Whatever drew people to have thyroid function tested at this Thyroid Awareness health fair, resulted in an enriched prevalence of thyroid disease. This health fair effect may be explained by people attending the health fair because of an increased concern for thyroid disease and desire to be tested. Such concerns may be multifactorial, perhaps reflecting an increased awareness of disease symptoms because of family history of thyroid disease, and/or the information presented by the media during Thyroid Awareness Week. Attracting people with a high proportion of suspect symptoms may contribute to the health fair effect in this study. The health fair also drew a greater proportion of women participants, and participants who are older than the general population. This contributed to the health fair effect by attracting a demographic known to have a higher likelihood of thyroid disease. Other unmeasured factors may also exist. Thus, diagnosing previously unknown thyroid dysfunction through testing of people who present to disease-specific health screenings may be both effective and appropriate. Trying to discriminate between individuals with and without thyroid dysfunction solely based on symptoms may be more appropriate in other settings, such as primary care clinics. Further studies are needed to look at the cost-effectiveness of thyroid screening in health fair populations, and populations that resemble health fair attendees, who have a greater likelihood of disease.

## Competing interests

In the past five years, the PI has not received reimbursements, fees, funding or salary from an organization that may in any way gain or lose financially from the publication of this manuscript either now or in the future. The data collected for this manuscript was obtained through a health fair, “Thyroid Awareness Week”, which was sponsored by St. Mary’s Health Services of Grand Rapids, Michigan, in conjunction with Boots Pharmaceutical Company. The PI’s time on the manuscript and the article-processing charge is supported by her current employer, the University of Nebraska Medical Center. The PI does not hold any stocks or shares in an organization that may in any way gain or lose financially from the publication of this manuscript, either now or in the future. The PI does not hold and is not applying for any patents relating to the content of the manuscript. The PI has not received reimbursement, fees, funding or salary from an organization that holds or has applied for patents relating to the content of the manuscript. The PI does not have any other financial competing interests, nor any non-financial competing interests that would be political, personal, religious, ideological, academic, intellectual, commercial or other to declare in relation to this manuscript.

## Authors’ contributions

GJC developed the thyroid symptoms questionnaire, conceived of the study, participated in its design and in the data collection, was involved in the interpretation of the data results and was primarily responsible for the drafting of the manuscript. TGT performed the statistical analysis and was involved in the interpretation of the data, and was involved in critically revising the manuscript for important intellectual content. RSW performed the statistical analysis and was involved in the interpretation of the data, and involved in critical revision of the manuscript for important intellectual content. All authors read and approved the final manuscript.

## Pre-publication history

The pre-publication history for this paper can be accessed here:

http://www.biomedcentral.com/1471-2458/13/351/prepub

## Supplementary Material

Additional file 1: Table S1 Health Fair participants’ symptom prevalence without significant difference; Prior Case-control euthyroid participants’ symptom prevalence for comparison [13]. Click here for file

Additional file 2: Table S2 Symptom prevalence in euthyroid and hypothyroid health fair participants, comparing those taking thyroid medication (previously diagnosed) and those not taking thyroid medication (new diagnosis through the health fair). Click here for file
