# Supplementary material for: Thyroid disease awareness is associated with high rates of identifying subjects with previously undiagnosed thyroid dysfunction
Source: BMC Public Health. 2013 Apr 16;13:351. doi: 10.1186/1471-2458-13-351 (PMC3643833; doi:10.1186/1471-2458-13-351)
Supplement: Additional file 1: Table S1 — Health Fair participants’ symptom prevalence without significant difference; Prior Case-control euthyroid participants’ symptom prevalence for comparison [13]. [file 1471-2458-13-351-S1.doc]

**Additional file 1:** **Table S1 Health Fair participants’ symptom prevalence without significant difference; Prior Case-control euthyroid participants’ symptom prevalence for comparison [13].**

| **Symptom present**  Symptom compared to  a year ago | **HEALTH FAIR hypothyroid persons reporting symptom**  **n / n respondents (%)** | **HEALTH FAIR euthyroid persons reporting symptom**  **n / n respondents (%)** | **Health Fair**  **p value** | **CASE- CONTROL euthyroid persons reporting symptom (%)** |
| --- | --- | --- | --- | --- |
| 1. **Hoarse voice** | 15/101 **(14.9)** | 84/627 **(13.4)** | 0.76 | 4 |
| Hoarser voice | 6/100 **(6.0)** | 63/627 **(10.0)** | 0.20 | 4 |
| 2. **Deep voice** | 14/102 **(13.7)** | 108/628 **(17.2)** | 0.50 | 8 |
| Deeper voice | 4/98 **(4.1)** | 27/620 **(4.4)** | 0.99 | 2 |
| 3. **Dry skin** | 70/100 **(70.0)** | 428/625 **(68.5)** | 0.65 | 54 |
| Drier skin | 27/99 **(27.3)** | 210/636 **(33.0)** | 0.23 | 24 |
| 4. **Coarse hair** | 12/102 **(11.8)** | 103/636 **(16.2)** | 0.30 | 14 |
| Coarser hair | 5/99 **(5.1)** | 30/636 **(4.7)** | 0.85 | 2 |
| 5. **Cold sensitive** | 47/97 **(48.5)** | 304/619 **(49.1)** | 0.87 | 40 |
| Colder | 11/100 **(11.0)** | 107/629 **(17.0)** | 0.14 | 11 |
| 6. **Tired** | 48/97 **(49.5)** | 358/634 **(56.5)** | 0.12 | 30 |
| More tired | 35/100 **(35.0)** | 255/638 **(40.0)** | 0.38 | 26 |
| 7. **Puffy eyes** | 37/99 **(37.4)** | 230/632 **(36.4)** | 0.82 | 17 |
| Eyes more puffy | 13/99 **(13.1)** | 110/636 **(17.3)** | 0.30 | 9 |
| 8. Sleep more | 40/101 **(39.6)** | 267/633 **(42.2)** | 0.88 | 31 |
| 9. **Muscle cramps** | 24/102 **(23.5)** | 162/641 **(25.3)** | 0.75 | 15 |
| Muscles cramp more | 19/101 **(18.8)** | 134/632 **(21.2)** | 0.71 | 16 |
| 10. **Weak muscles** | 30/100 **(30.0)** | 205/629 **(32.6)** | 0.61 | 21 |
| Weaker muscles | 25/100 **(25.0)** | 151/635 **(23.8)** | 0.76 | 18 |
| 11. **Constipated** | 16/102 **(15.7)** | 139/641 **(21.7)** | 0.18 | 10 |
| Constipat’d more often | 8/102 **(7.8)** | 60/637 **(9.4)** | 0.58 | 6 |
| 12. **Depressed** | 21/95 **(22.1)** | 191/620 **(30.8)** | 0.06 | 12 |
| More depressed | 23/100 **(23.0)** | 176/635 **(27.7)** | 0.37 | 18 |
| 13. **Slow thinking** | 22/97 **(22.7)** | 180/622 **(28.9)** | 0.16 | 10 |
| Slower thinking | 26/100 **(26.0)** | 200/637 **(31.4)** | 0.29 | 14 |
| 14. **Poor memory** | 17/99 **(17.2)** | 157/628 **(25.0)** | 0.08 | 16 |
| Poorer memory | 23/101 **(22.8)** | 202/635 **(31.8)** | 0.07 | 15 |
| 15. **Math difficulty** | 18/98 **(18.4)** | 94/640 **(14.7)** | 0.42 | 11 |
| Math more difficult | 12/100 **(12.0)** | 81/639 **(12.7)** | 0.73 | 4 |
| 16. **Irregular menses** | 8/25 **(32.0)** | 76/226 **(33.6)** | 0.87 | 29 |
| Menses more irregular | 5/25 **(20.0)** | 52/227 **(22.9)** | 0.74 | 27 |
| 17. **Heavy menses** | 8/25 **(32.0)** | 77/219 **(35.2)** | 0.75 | 29 |
| Heavier menses | 5/25 **(20.0)** | 43/225 **(19.1)** | 0.87 | 26 |
