# Supplementary material for: Thyroid disease awareness is associated with high rates of identifying subjects with previously undiagnosed thyroid dysfunction
Source: BMC Public Health. 2013 Apr 16;13:351. doi: 10.1186/1471-2458-13-351 (PMC3643833; doi:10.1186/1471-2458-13-351)
Supplement: Additional file 2: Table S2 — Symptom prevalence in euthyroid and hypothyroid health fair participants, comparing those taking thyroid medication (previously diagnosed) and those not taking thyroid medication (new diagnosis through the health fair). [file 1471-2458-13-351-S2.doc]

**Additional file 2: Table S2 Symptom prevalence in euthyroid and hypothyroid health fair participants,** comparing those taking thyroid medication (previously diagnosed) and those not taking thyroid medication (new diagnosis through the health fair).

| **Symptom present**  Symptom compared to a  year ago | **Taking thyroid medication:**  **% reporting symptom** | | **Not**  **taking thyroid medication:**  **% reporting symptom** | | **Hypothyroid**  **p value** | **Euthyroid p value** |
| --- | --- | --- | --- | --- | --- | --- |
|  |  | |  | |  |  |
|  | **HYPO** | **EU** | **HYPO** | **EU** |  |  |
| 1. **Hoarse voice** | 12.0 | 21.8 | 15.8 | 12.6 | 0.64 | 0.05 |
| Hoarser voice | 4.0 | 5.4 | 6.7 | 10.5 | 0.63 | 0.22 |
| 2. **Deep voice** | 20.0 | 18.5 | 11.7 | 17.1 | 0.29 | 0.79 |
| Deeper voice | 4.0 | 0.0 | 4.1 | 4.8 | 0.98 | 0.11 |
| 3. **Dry skin** | 68.0 | 83.9 | 70. 7 | 67.0 | 0.80 | 0.01 |
| Drier skin | 20.0 | 28.1 | 29.7 | 33.5 | 0.34 | 0.40 |
| 4. **Coarse hair** | 16.0 | 12.7 | 10.4 | 16.5 | 0.45 | 0.47 |
| Coarser hair | 8.0 | 0.0 | 4.0 | 5.2 | 0.44 | 0.08 |
| 5. **Cold sensitive** | 45.8 | 70.9 | 49.3 | 47.0 | 0.77 | 0.001 |
| Colder | 4.0 | 27.8 | 13.3 | 16.0 | 0.20 | 0.03 |
| 6. **Tired** | 50.0 | 61.4 | 49.3 | 56.0 | 0.95 | 0.43 |
| More tired | 37.5 | 33.3 | 34.2 | 40.6 | 0.77 | 0.28 |
| 7. **Puffy eyes** | 29.2 | 37.0 | 40.0 | 36.3 | 0.34 | 0.92 |
| Eyes more puffy | 8.3 | 10.9 | 14.7 | 17.9 | 0.42 | 0.19 |
| 8. Sleep more | 52.0 | 47.4 | 35.5 | 41.7 | 0.14 | 0.41 |
| 9. **Muscle cramps** | 20.0 | 19.3 | 24.7 | 25.9 | 0.63 | 0.28 |
| Muscles cramp more | 20.0 | 12.3 | 18.4 | 22.1 | 0.86 | 0.08 |
| 10. **Weak muscles** | 28.0 | 36.8 | 30.7 | 32.2 | 0.80 | 0.47 |
| Weaker muscles | 12.0 | 24.6 | 29.3 | 23.7 | 0.08 | 0.88 |
| 11. **Constipated** | 16.0 | 33.9 | 15.6 | 20.5 | 0.96 | 0.02 |
| Constipated more often | 4.0 | 7.3 | 9.1 | 9.6 | 0.41 | 0.57 |
| 12. **Depressed** | 28.6 | 33.9 | 20.3 | 30.5 | 0.42 | 0.60 |
| More depressed | 26.1 | 17.9 | 22.1 | 28.7 | 0.69 | 0.08 |
| 13. **Slow thinking** | 25.0 | 50.9 | 21.9 | 26.8 | 0.75 | 0.001 |
| Slower thinking | 16.7 | 33.3 | 28.9 | 31.2 | 0.23 | 0.74 |
| 14. **Poor memory** | 17.4 | 41.8 | 17.1 | 23.4 | 0.97 | 0.003 |
| Poorer memory | 12.5 | 30.9 | 26.0 | 31.9 | 0.17 | 0.88 |
| 15. **Math difficulty** | 9.1 | 17.5 | 21.0 | 14.4 | 0.20 | 0.52 |
| Math more difficult | 4.4 | 7.0 | 14.3 | 13.2 | 0.20 | 0.18 |
| 16. **Irregular menses** | 60.0 | 18.2 | 25.0 | 34.4 | 0.33 | 0.43 |
| Menses more irregular | 20.0 | 18.2 | 20.0 | 23.1 | 0.53 | 0.99 |
| 17. **Heavy menses** | 16.7 | 36.4 | 36.8 | 35.1 | 0.67 | 0.81 |
| Heavier menses | 16.7 | 27.3 | 21.1 | 18.7 | 0.73 | 0.75 |
